# Sputum Smear Positive Pulmonary Tuberculosis Diagnostic Dropout Rate in Public Health Facilities, Addis Ababa, Ethiopia

**DOI:** 10.1155/2019/2905615

**Published:** 2019-03-20

**Authors:** Daniel Melese Desalegn, Kumera Terfa Kitila, Boja Dufera Taddese, Tinsae Kidanemariam Hailu, Tariku Takle Dinku, Kassahun Demisse Asferie, Hanna Mekonnen Balcha, Chalachew Sisay Gebeyehu, Girmay Medhin

**Affiliations:** ^1^Ethiopian Public Health Institute (EPHI), Addis Ababa, Ethiopia; ^2^Addis Ababa Public Health Research and Emergency Management Core Process, Addis Ababa City Administration Health Bureau, Addis Ababa, Ethiopia; ^3^Aklilu Lemma Institute of Pathobiology Addis Ababa University, Addis Ababa, Ethiopia

## Abstract

**Background:**

Prolonged laboratory diagnostic process of tuberculosis can lead to failure to complete the diagnosis and increase dropout rate of smear positive pulmonary tuberculosis (PTB) cases. This implies such dropout patients without completing diagnosis are critical as infected individuals remain untreated in the community, providing more opportunities for transmission of the disease and adversely affecting the epidemic. The aim of this research is to determine the level of smear positive PTB diagnosis dropout rate of spot-morning-spot sputum microscopy diagnosis method in public health facilities, in Addis Ababa, Ethiopia.

**Methods:**

Retrospective review of patient documents in 13 public health facilities' TB laboratory in Addis Ababa was conducted from October 2011 to March 2016. Data was computerized using Epi-info software and analysed using SPSS version 20.0 software. Descriptive numerical summaries were used to present the findings. Association between the dropout rate and demographic variables was assessed by Chi-square (X^2^). Bivariate model using Odds Ratio (OR) with a 95% Confidence Interval (CI) was calculated. P-Value less than 0.05 was taken as statistically significant.

**Results:**

Of 41,884 presumptive TB patients registered during the 53 months for laboratory investigation, 5.9% were positive for the first spot sputum smear microscopy. Among these positive cases, 142 (5.8%) and 298 (12.1%) did not come back to the laboratory to submitted early morning and second spot sputum specimens, respectively. The diagnostic dropout for morning sputum specimen in hospitals was 5.6% (58/1039) and in health centres was 5.9% (84/1424). However, higher proportion of dropout for second spot sputum specimen in hospitals was 16.4% (170/1039), compared to the health centres, 8.9% (128/1424). Diagnostic dropout of sputum smear microscopy had no significant association with sociodemographic variable (P value >0.05), while it had significant association with facility type (P value <0.05).

**Conclusion:**

In this study smear positive pulmonary tuberculosis diagnostic dropout rate was high compared to WHO reported for the new strategy shift implying the importance of shifting to same-day approach. Hence, shifting from conventional to same day is crucial to minimize the TB diagnostic dropout rate in the study area and other similar settings. Further research is needed/recommended in the local setting to compare the yield and dropout rates between same-day and conventional sputum smear microscopy approach.

## 1. Background

Sputum smear microscopy diagnosis is one of the key pillars of strategies for tuberculosis control. Unfortunately, the smear positivity depends on time of sputum collection, the number specimens to be examined, and the load of bacilli in specimen [1]. As a result, TB control program recommended spot-morning-spot (SMS) to increase the positivity yields of sputum smears examine. However, it may be costly and inconvenient for patients who have to make multiple visits to health facilities to submit three sputum specimens over several days [2–4]. This approach can prolong the diagnostic process of TB suspected individuals, which may lead to dropout of TB patients from diagnostic paths [1].

Smear positive PTB diagnostic dropout cases are more common in TB prevalent setting. Studies conducted in Africa at different setting showing 6 to 38% TB cases failed (dropped out) from diagnostic pathway (loss to follow-up during diagnostic period) [2]. Systematic review and meta-analysis has shown that patients assigned to same-day diagnosis were more likely to submit both specimens (dropout= 2%) than patients screened conventionally (dropout=5.8%) [4]. Hence, to minimize the dropout, WHO has recently recommended a reduction in the number of specimens examined from three to two in settings with appropriate external quality assurance systems of smear microscopy being in place and good-quality microscopy results have been documented [4, 5]. Operational research is needed to ensure the quality assurance based on the country-specific evidence in terms of accuracy, diagnostic dropout rate, and external quality assurance systems of smear microscopy before switching to same-day diagnosis. However, the strength of quality assurance system is questionable and TB diagnostic dropout is unknown in our setting. Therefore, this study was designed to determine the level of smear positive PTB diagnostic dropout rate of spot-morning-spot (SMS) sputum microscopy diagnosing method in public health facilities in Addis Ababa, Ethiopia, with the potential to generalize the findings to the whole country and beyond.

## 2. Methods

### 2.1. Study Design and Setting

Retrospective patient record review was conducted in Addis Ababa city covering the period from October 2011 to March 2016. Addis Ababa city Administration has a population of 3,384,569, with annual growth rate of 3.8% [6]. Health care facility expansion has improved physical access to health services with an emphasis on primary health care units, resulting in potential health service coverage of Addis Ababa to an estimated 100%. The city has 47 hospitals, 204 higher, 226 medium, 143 lower private clinics, and 100 public health centres [7]. Regarding TB diagnostic services, at the time of this assessment, all private hospitals, higher clinics, and all public health facilities were providing TB diagnostic and treatment services.

### 2.2. Study Subjects

Thirteen health facilities' laboratories (10 health centres and 3 hospital laboratories) providing tuberculosis diagnostic and treatment service and those which have records of Ziehl-Neelsen (ZN) stained smear microscopy resulting from October 2011 to March 2016 were included in the current study. Individual patients data documented in the TB record book of these laboratories were used as study subjects.

### 2.3. Data Sources and Sampling

Health institutions were selected using simple random sampling with the plan of obtaining three hospitals from six public hospitals and ten health centres (one from each subcity). The health facilities' sputum smear microscopy records, from a period of 2011 to 2016, were used as data source. Status of the record was assessed during reviews of records.

### 2.4. Inclusion and Exclusion Criteria

All PTB cases having complete records in the laboratory, from the years 2011 to 2016, were included. However, follow-up case, any form of TB other than PTB, TB laboratory results performed by other than ZN technique, and incomplete records were excluded from analysis.

### 2.5. Data Management and Statistical Analysis

The collected data were checked for completeness and consistency and computerized using Epi-info software. The data were coded and analysed using SPSS version 20.0 (SPSS Inc., Chicago, USA) software. Descriptive numerical summaries were used to present the findings of sociodemographic characteristics and trends of pulmonary tuberculosis diagnostic dropout rate. Association between the dropout rate and demographic variables was assessed by Chi-square (X^2^). Bivariate model using Odds Ratio (OR) with a 95% Confidence Interval (CI) was calculated after excluding missing data to identify factors associated with the smear positive PTB diagnostic dropout rate. P-value less than 0.05 was taken as statistically significant.

### 2.6. Data Quality Assurances

Before extracting data from records, data collectors were adequately trained and they were instructed to check the completeness of each data before submission. Quality of data collection process was supervised and monitored by the principal investigator.

### 2.7. Operational Definition

*Diagnosis dropout*: it is defined as the patient does not turn back to submit the morning and/or the second spot sputum specimens, while their first sputum examination is positive.

*Conventional (two-day) sputum microscopy diagnostic approach*: TB suspect patient to give three sputum specimens (SMS) within two consecutive days to diagnosis TB.

*Same-day sputum microscopy diagnostic approach*: a new sputum microscopy diagnosis algorithm requires a TB suspect patient to give two sputum specimens (SS) within the same day to diagnosis TB.

## 3. Result

### 3.1. Sociodemographic Characteristics

During the target period 41,884 presumptive TB patients were registered for sputum smear microscopy examination in the study health facilities. Among these patients 2,463 were positive for the first spot sputum samples. Of these first spot positive TB cases, 1,291(52.4%) were males, 70(2.8%) were below the age of 15 years, and 594(24.1%) were above 35 years. Records were incomplete for 194(7.8%) on patients age and 95(3.9%) on patient sex [Table 1].

### 3.2. Diagnostic Dropout Rate of Smear Positive PTB

Of 41,884 presumptive TB cases recorded in the laboratory registration book in the period from the year 2011-2016, 2,463(5.9%) were smear positive for the first spot sputum samples. Among 2,463 first spot smear positive TB cases, 142 (5.8%) and 298(12.1%) did not get back (dropout) to submit morning and second spot sputum specimen, respectively. Overall sputum smear positive diagnosis dropout rate was 17.9% (440/2463); no patients had dropped out for both sputum (the morning and the second pot). The diagnostic dropout rate for morning sputum positive specimen in hospitals was 5.6% (58/1,039) and in health centres was 5.9% (84/1,424). However, higher proportion of dropout rate for second spot sputum positive specimen in hospitals was 16.4% (170/1,039) compared to the health centres, 9.0% (128/1,424). Percentage dropout for morning positive specimen was 5.7% (4/70) in the younger age group (i.e., below the age of 15 years) and 5.6% (33/594) in the older age group (i.e., above 35 years of age). Similarly, percentage dropout for second spot positive specimen was 7.1% (5/70) in the younger age group and 12.8% (76/594) in the older age group. Diagnostic dropout rate for morning positive specimen was 72/1,291(5.6%) among males and 60/1,077(5.6%) among females. The dropout rate for second spot positive specimen was 154/1,291(11.9%) among males and 126/1,077(11.7%) among females [Table 2].

### 3.3. Trends of Diagnostic Dropout Rate of Smear Positive PTB

**Figure 1:**
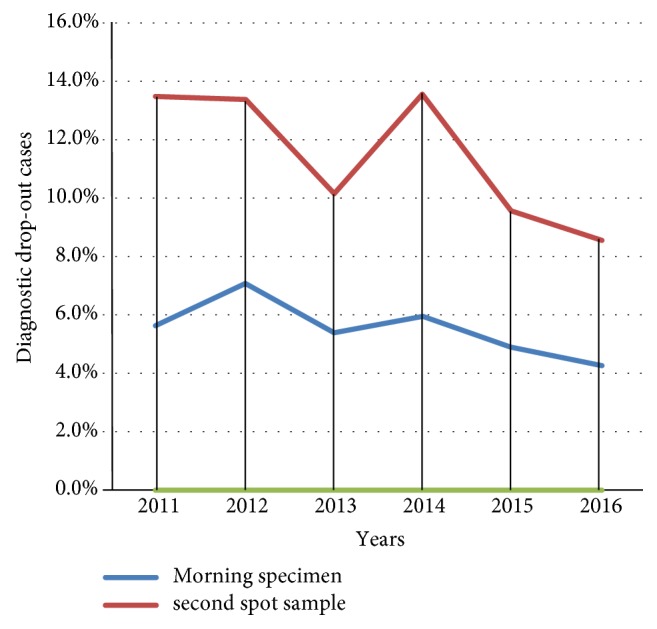
Trends of sputum smears positive pulmonary tuberculosis diagnostic dropout rate from 2011-2016.

Among 142 TB diagnostic dropouts for morning specimen, 31/540(5.7%), 35/491(7.1%), 24/440(5.4%), 26/434(6.0%), 17/348(4.9%), and 9/210(4.3%) were in the years 2011, 2012, 2013, 2014, 2015, and 2016, respectively. Whereas among 298 TB diagnostic dropouts for second spot, 73/540(13.5%), 66/491(13.4%), 45/440(10.2%), 59/434(13.6%), 37/348(9.6%), and 18/210(8.6%) were in the years 2011, 2012, 2013, 2014, 2015, and 2016, respectively [Figure 1].

In mono-variate analysis, the trends of diagnostic dropout of sputum smear microscopy had no significant changes among the years from 2011 to 2016 (P value >0.05), even though the number of patients diagnostic dropout becomes slightly decreasing to the year 2016 (Table 3).

### 3.4. Factors Affecting Sputum Smear Positive PTB Diagnostic Dropout

In this study diagnostic dropout of sputum smear microscopy had no statistically significant association with sociodemographic variables (P value >0.05), while diagnostic dropout had significant association with facility type (P value < 0.05) (Table 4).

## 4. Discussion

From the total presumptive TB cases registered for sputum smear microscopy diagnosis, the positivity rate for first spot specimens was 5.9%. Among these first spot positive cases, 17.9% did not come back to the laboratory to submit second and/or third specimens on the next day visit. The diagnostic dropout rate in Addis Ababa was higher than the number that WHO reported. The higher dropout rate was associated with the second sputum spot collection and related to hospital services in comparison to health centres. The diagnostic dropout rate was increased by ages but not significantly.

In the current study smear positivity rate was lower than the previous report of 14.2% in Metehara Sugar Factory Hospital, 9.2% in South East Ethiopia, and 10.4% in North Gondar Zone [8–10]. However, it was comparable to the 6.1% reported previously in Mwanza, Tanzania [11]. Similar studies indicated that sputum positivity rate depended on different factors including patients' factors, sputum sample quality, and laboratory diagnostic capacity [12, 13]. Improving TB diagnostic capacity, staining quality, sample collection, and adherence to the national TB laboratory standards is crucial to increase smear positivity [12, 13].

Diagnostic dropout rate for second spot sputum specimen in the current study was higher than 5.8% reported by WHO for new strategy shift, 5% reported in Pakistan, and 10.2% reported in Botswana [4, 14, 15]. It was comparable to the finding of 13% reported in Chennai of India [16]. It was lower than studies which reported 52.0% in South Africa, 37.6% in Lilongwe of Malawi, and 38.0% in Ghana [17–19]. The difference might be due to health-system-related factors including delays in getting the results of sputum smears microscopy diagnosis and tracing mechanism of dropout cases. Patient related factors including understanding the nature of the disease, its severity, and potential benefits of early diagnosis and treatment initiation might vary among different countries. In addition, the way health workers treat patients (handling systems) might be as factors for TB diagnostic dropout. This clearly indicates that, besides shifting from conventional to same-day TB diagnostic approach, there is need to work on different factors to minimize diagnostic dropout rate in the study area and others similar setting.

In this study diagnostic dropout rate for second spot sputum specimen in hospital was high as compared to the health centres. Studies conducted in Botswana and South Africa have also reported high dropout rate in hospital [15, 20]. The possible reasons for this can be that customers might perceive that hospital has better services which might cause additional workload in hospitals, resulting in long waiting time, delays in receiving results, and patients' dissatisfaction which might contribute to increasing dropout in hospital. In addition, accessibility of health centres might contribute to the difference. The health centres coverage in Addis Ababa is near 100% and easily accessible and does not incur much cost for patients to summit second and third specimens on the next day visit, which might reduce dropout in health centres.

In this study diagnostic dropout rate in the age ranges of 15-35 years was lower than 39.2% reported from Vietnam among age group of 15–34 years [21]. The diagnostic dropout rate for second spot specimen among male was lower compared to the study which reported 70.5% in Vietnam [21]. In this study diagnostic dropout rate was higher among males compared to females. Study of Vietnam and South India also reported high drop out among male than female [21, 22]. These findings point toward the need to further study age and sex differences in TB diagnostic dropout rate in order to inform age and sex specific diagnostic defaulter prevention.

## 5. Strengths and Limitations of the Study

This is a study based on a very large size retrospective patient's record review to determine the conventional sputum smears microscopy diagnostic dropout rate. The result may be used as an input for the national TB control program by providing reliable and accurate conclusions implying the importance of shifting to same-day approach. However, the findings are not without limitations. Results are dependent only on the record review, despite some demography data which was incomplete in the laboratory log books. In addition this study was conducted only in public health facilities and hence does not necessarily illustrate the situation in private health situations.

## 6. Conclusion and Recommendations

In this study smears positive PTB diagnostic dropout rate of conventional sputum microscopy was highly compared to WHO reported for the new strategy shift implying the importance of shifting to same-day approach. The results from this investigation reinforce the importance that patients should promptly seek the appropriate medical attention following onset of TB symptoms. This study indicated that a high number of presumptive TB cases failed to submit morning and second spot specimen for laboratory investigation; as a result, a high number of active TB cases were untreated and might be potential source of TB transmission to the community, resulting in potential TB transmission to their close contacts, including those caring for the sick person at the later stages of the disease and with the ever increasing threat of MDR and XDR-TB as well as HIV. Hence, shifting from conventional to same-day sputum microscopy is crucial to minimize the TB diagnostic dropout rate in the study area and others similar setting. Research is needed in the local setting to compare the yield and dropout rates between same-day and conventional sputum smear microscopy approach.

## Figures and Tables

**Table 1:** Socio demographic characteristics of Smears positive PTB diagnostic dropout rate at public health facilities in Addis Ababa, Ethiopia, from 2011-2016.

Variables		Number	Percent
Age group in year	<15	70	2.8
	15-18	170	6.9
	19-35	1435	58.3
	36-55	456	18.5
	>55 y	138	5.6
	Data missing	194	7.9
	*Total*	*2463*	*100*
Sex	Male	1291	52.4
	Female	1077	43.7
	Data missing	95	3.9
	*Total*	*2463*	*100*

**Table 2:** Mono-variate analysis the association between dropout rate and age, sex, health facility and period of registration of sputum smear positive PTB at public health facilities in Addis Ababa, Ethiopia, from 2011-2016.

Independent variables	First sputum AFB positive	Not submit the morning spot(a)				Not submit the 2nd spot (b)				Not submit the morning or the 2nd spots (a+b)				p –values
		n	%	OR	95%CI	n	%	OR	95%CI	n	%	OR	95%CI	
*Total*	*2463*	*142*	*5.8%*			*298*	*12.1%*			*440*	*17.9%*			
*Age in year*														
< 15yr.	70	4	5.7%	1.03	0.35-3.00	5	7.1%	0.52	0.20-1.34	9	12.9%	0.66	0.32-1.36	0.236
15 - 35yr.	1605	90	5.6%	1.01	0.69-1.49	182	11.3%	0.87	0.65-1.16	272	16.9%	0.91	0.71-1.16	
> 35yr.	594	33	5.6%	1		76	12.8%	1		109	18.4%	1		
*Missing*	194	15	7.7%			35	18.0%			50	25.8%			
*Sex*														
Male	1291	72	5.6%	1		154	11.9%	1		226	17.5%	1		
Female	1077	60	5.6%	1	0.7-1.42	126	11.7%	0.98	0.76-1.26	186	17.3%	0.98	0.79-1.22	
*Missing*	95	10	10.5%			18	18.9%			28	29.5%			
*Health facilities*														
Hospital	1039	58	5.6%	0.94	0.62-1.33	170	16.4%	1.98	1.55-2.53	228	21.9%	1.61	1.31-1.98	<0.001^*∗*^
Health Center	1424	84	5.9%	1		128	9.0%	1		212	14.9%	1		
*Year of sputum submission*														
2011	540	31	5.7%	1		73	13.5%	1		104	19.3%	1		
2012	491	25	7.1%	1.26	0.76-2.08	66	13.4%	0.99	0.69-1.42	101	20.6%	1.09	0.80-1.47	0. 362
2013	440	24	5.5%	0.95	0.55-1.64	45	10.2%	0.73	0.49-1.08	69	15.7%	0.78	0.56-1.09	
2014	434	26	6.0%	1.05	0.61-1.79	59	13.6%	1.01	0.70-1.46	85	19.6%	1.02	0.74-1.41	
2015	348	17	4.9%	0.84	0.46-1.55	37	10.6%	0.80	0.50-1.09	54	15.5%	0.79	0.57-1.09	
2016	210	9	4.3%	0.73	0.34-1.57	18	8.6%	0.81	0.51-1.09	27	12.9%	0.83	0.61-1.31	

*Key*: ^*∗*^statistically significant, *P<0.00*.

**Table 3:** Mono-variate analysis the association between dropout and registration of sputum submission period at public health facilities in Addis Ababa, Ethiopia, from 2011-2016.

Independent variables	First AFB positive	Not submit the morning spot(a)				Not submit the 2nd spot (b)				Not submit the morning or the 2nd spots (a+b)				p –value
		n	%	OR	95%CI	n	%	OR	95%CI	n	%	OR	95%CI	
*Total*	*2463*	*142*	*5.8%*			*298*	*12.1%*			*440*	*17.9%*			
Year of sputum submission														
2011-2013	1471	90	6.1%	1		184	12.5%	1		274	18.6%	1		0.229
2014-2016	992	52	5.2%	0.85	0.61-1.21	59	5.9%	0.91	0.71-1.17	166	11.2%	0.88	0.71-1.09	

**Table 4:** Determinants on sputum smears positive pulmonary tuberculosis diagnostic dropout rate in public health facilities, in Addis Ababa, Ethiopia; from 2011 to 2016.

Variables	Drop out of diagnostic			Crude Odds		P-value
	Yes	No	%Yes	Ratio	95%CI	
*Age groups (years)*						
<15	9	61	12.9*%*	1		
15-35	272	1333	16.9*%*	1.32	0.71-2.45	0.380
35+	109	485	18.4*%*	1.43	0.76-2.69	0.258
*Sex*						
Male	226	1065	17.5*%*	1		
Female	186	891	17.3*%*	0.99	0.83-1.18	0.881
*Facilities*						
Hospital	228	811	21.9*%*	1		
Health Center	212	1212	14.9*%*	0.62	0.51-0.77	<0.001^**∗**^

^**∗**^Statistically high significant, P<0.005.

## Data Availability

The data used to support the finding of this study cannot be shared in a publically available data repository system, because there is no such data repository system in the country. However, the data are available from the authors upon request at any time.

## Abbreviations

AFB: Acid Fast Bacillus (or Bacilli)
EQA: External quality assessment
SMS: Spot-morning-spot
TB: Tuberculosis
PTB: Pulmonary tuberculosis
WHO: World Health Organization
ZN: Ziehl-Neelsen stain.
